# ggpedigree: Visualizing Pedigrees with ‘ggplot2’ and ‘plotly’

**DOI:** 10.21105/joss.09434

**Published:** 2026-02-01

**Authors:** S. Mason Garrison

**Affiliations:** 1 Department of Psychology, Wake Forest University, North Carolina, USA

## Abstract

Pedigree diagrams underpin research and practice across genetics, animal breeding, genealogy, forensics, and counseling. They help medical geneticists trace the inheritance of Mendelian diseases and identify at-risk relatives; enable dairy breeders to plan matings that improve milk yield; support genealogists in reconstructing ancestry; assist forensic scientists in establishing familial connections in criminal investigations; and facilitate family therapists and counselors in understanding their clients’ relationships (McGoldrick, Gerson, & Petry, 2020). Early R tools such as kinship2 (Sinnwell, Therneau, & Schaid, 2014) plot simple nuclear families effectively, but they do not scale to today’s pedigrees that can exceed 1,000s of individuals. As datasets have grown, researchers now work with increasingly complex family structures, including large-scale plant breeding pedigrees (Shaw, Graham, Kennedy, Milne, & Marshall, 2014), web-based pedigree management systems (Ranaweera, Makalic, Hopper, & Bickerstaffe, 2018), interactive pedigree editors (Carver et al., 2018), and behavior genetic studies of extended family structures (Garrison et al., 2023; Hunter, Garrison, Burt, & Rodgers, 2021). That complexity exposes the limitations of existing tools, which often struggle with large and complex datasets. ggpedigree addresses this need by combining a vectorised layout algorithm, ggplot2 output, and optional plotly interactivity.

## Statement of need

Pedigree visualization has traditionally relied on proprietary software (e.g., Progeny, GenoPro, Pedigree Viewer) or R packages like kinship2 (Sinnwell et al., 2014), pedtools (Vigeland, 2021a), or pedtricks (J. Martin, Wolak, Johnston, & Morrissey, 2025). While these tools are functional for many use cases, their limitations become pronounced when working with complex, modern pedigree datasets or when more detailed customization is required. Most R packages focus on base graphics or simple ggplot2 implementations.

Existing R solutions face three main challenges. First, current solutions are often poorly integrated with tidyverse workflows and do not expose the full theming and layering capabilities familiar to ggplot2 users (Wickham, 2016). In animal-focused workflows, rapid rendering seems to takes precedence over aesthetic flexibility. I suspect that this is because users tend to work with more uniform data and fewer phenotypes. By contrast, human-focused workflows—particularly in behavior genetics and genetic epidemiology (Garrison et al., 2023; Lyu et al., 2025; McArdle & McDonald, 1984)—require closer integration with tidyverse pipelines and more flexible plotting systems to accommodate complex pedigree structures and harmonization of phenotypes across data sources. In other words, the needs are different.

Second, most R-based tools offer no interactivity. Static graphics are often sufficient for publication, but interactivity improves exploration and communication during model development or data cleaning. A notable exception is pedtools (Vigeland, 2021b), which offers a sister shiny app, QuickPed (Vigeland, 2022). While the R ecosystem includes libraries, like plotly, that support interactive plotting, these features have yet to be integrated into pedigree functions.

Third, scalability and extensibility remain limited across existing tools. Several R packages attempt to address these challenges with built-in pedigree plotting functions. kinship2 (Sinnwell et al., 2014) remains widely used but produces static base graphics and relies on non-vectorized recursive layout functions that do not scale well to large families. A partial ggplot2 implementation exists in a modernized kinship2 (called Pedixplorer, Le Nézet, Sinnwell, Letko, André, & Quignon, 2025), but is non-vectorized and incompatible with other ggplot2 layers. pedtricks, a revival of pedantics (Morrissey & Wilson, 2010), provides a ggplot2-based implementation for large animal pedigrees but lacks extensibility and interactivity. The geneHapR (Zhang, Jia, & Diao, 2023) package focuses on haplotype visualization rather than general pedigree structure. The pedgene package (Schaid & Sinnwell, 2024) offers some plotting functions but is primarily designed for association testing. The pedigreejs package (Carver et al., 2018) provides an interactive pedigree editor but does not integrate with R or ggplot2, limiting its utility for R users.

None of these packages offers the combination of modern ggplot2 integration, interactive capabilities, and extensibility that ggpedigree provides. ggpedigree addresses these limitations by providing a comprehensive visualization framework built on modern R graphics infrastructure. It leverages the extensive customization capabilities of ggplot2 while adding specialized functionality for pedigree-specific visualization challenges.

## Software Architecture

ggpedigree is built on a modular architecture that separates data processing, layout calculation, and visualization layers. The core workflow involves: (1) data standardization and family restructuring using BGmisc functions, (2) coordinate calculation using algorithms adapted from kinship2, (3) relationship connection mapping, and (4) layer-based plot construction using ggplot2 geometry functions. This design allows users to customize any aspect of the visualization while maintaining computational efficiency for large pedigrees. The package integrates tightly with the broader R ecosystem, particularly the tidyverse (Wickham et al., 2019) and BGmisc (Garrison, Hunter, Lyu, Trattner, & Burt, 2024). All functions return standard R objects (ggplot or plotly) that can be further customized.

BGmisc, as described in Garrison et al. (2024), is a dependency for its relatedness-heavy workflows, supplying network-based validation utilities (checkParentIDs()) and relatedness components, like ped2fam(), ped2paternal(), and ped2maternal(). These components allow ggpedigree to visualize how related any two individuals are based on additive genetic, mitochondrial, or other relationship matrices. Burt et al. (2025) uses these features to create mitochondrial lineages in human pedigrees (n >176 million), finding that mitochondrial DNA explains significant variance in longevity.

## Features

I describe the main features of the ggpedigree package below. Detailed descriptions of features and usage are available in the package vignettes, including how to create static and interactive pedigree plots, customize aesthetics, and visualize relatedness matrices. Additional example data include squirrels from the Kluane Red Squirrel Project (McFarlane et al., 2014, 2015) and Targaryens from the Song of Ice and Fire universe (G. R. R. Martin, 1997, 2018).

- Data Standardization and Family Structure Analysis: ggPedigree() integrates with network-based functions from BGmisc like ped2fam() to organize individuals by family and checkParentIDs() to validate pedigree structures. The function handles consanguineous relationships and individuals appearing in multiple pedigree positions. More details are in the complex pedigree data vignette, as well as in these works (Garrison et al., 2024; Hunter et al., 2025, 2021).
- Coordinate Calculation: calculateCoordinates() computes optimal positioning for individuals using algorithms adapted from kinship2::align.pedigree, with enhancements for complex multi-generational pedigrees. These steps are vectorized as much as possible to ensure efficient computation and compatibility with ggplot2.
- Relationship Connection Mapping: calculateConnections() generates connection paths between family members, mapping parent-child, sibling, spousal, and twin relationships. The function determines midpoints for line intersections and handles overlapping connections with curved segments. These calculations are optimized for large datasets by using vectorized operations rather than the loop-based approaches used in kinship2.
- Layer-based Plot Construction: ggPedigree() constructs plots using ggplot2 geometry functions, returning standard ggplot2 objects that integrate with existing R workflows. ggPedigreeInteractive() extends plots into interactive plotly widgets. A config system allows customization of over 150 aesthetic and layout parameters. More details are in the configuration vignette.
- Individual Highlighting: Advanced functionality to highlight specific individuals and their relatives based on additive genetic, mitochondrial, or other relationship matrices.
- Specific Visualization Functions: ggPedigree() creates static pedigree plots using ggplot2. ggPedigreeInteractive() generates interactive pedigree plots using plotly. ggRelatednessMatrix() creates customizable heatmaps for relatedness matrices with support for hierarchical clustering, and seamless integration with BGmisc relatedness calculations. ggPhenotypeByDegree() visualizes phenotypic correlations as a function of genetic relatedness, including confidence intervals and statistical summaries for quantitative genetic analysis.

## Code example

This example shows how to use ggpedigree to visualize a pedigree. The potter dataset includes several wizarding families from the Harry Potter series.


ggPedigree(potter,
  famID = "famID",
  personID = "personID"
)


This code produces the following pedigree plot:


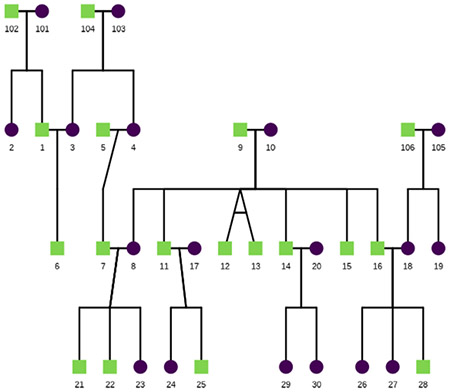


The package supports extensive customization of visual aesthetics. The following example is a figure from Hunter et al. (2025) that used the Potter pedigree data. The figure has been restyled according to Wake Forest University brand identity guidelines to demonstrate ggpedigree’s customization capabilities. The figure combines two panels: panel (a) highlights unique mitochondrial lines in the Dursley and Evans families, while panel (b) shows the full pedigree with Molly Weasley’s mitochondrial descendants in gold.


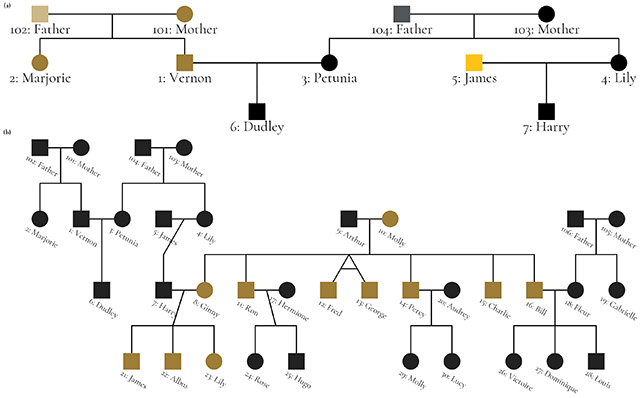


The complete source code for this example is available in the package documentation website.

Collectively, these tools provide a valuable resource for those work with extended family data. They were developed as part of a grant and have been used in several ongoing projects, presentations (Garrison, 2024; Hunter, Garrison, Lyu, Good, & Burt, 2024), and forthcoming papers (Burt et al., 2025; Hunter et al., 2025; Lyu et al., 2025).
